# Reduced activity of adenylyl cyclase 1 attenuates morphine induced hyperalgesia and inflammatory pain in mice

**DOI:** 10.3389/fphar.2022.937741

**Published:** 2022-09-02

**Authors:** Kayla Johnson, Alexis Doucette, Alexis Edwards, Aleeya Verdi, Ryan McFarland, Shelby Hulke, Amanda Fowler, Val J. Watts, Amanda H. Klein

**Affiliations:** ^1^ Department of Pharmacy Practice and Pharmaceutical Sciences, University of Minnesota, Duluth, MN, United States; ^2^ Department of Medicinal Chemistry and Molecular Pharmacology, Purdue University, West Lafayette, IN, United States

**Keywords:** pain, adenylyl cyclase, tolerance, hypersensitivity, inflammation

## Abstract

Opioid tolerance, opioid-induced hyperalgesia during repeated opioid administration, and chronic pain are associated with upregulation of adenylyl cyclase activity. The objective of this study was to test the hypothesis that a reduction in adenylyl cyclase 1 (AC1) activity or expression would attenuate morphine tolerance and hypersensitivity, and inflammatory pain using murine models. To investigate opioid tolerance and opioid-induced hyperalgesia, mice were subjected to twice daily treatments of saline or morphine using either a static (15 mg/kg, 5 days) or an escalating tolerance paradigm (10–40 mg/kg, 4 days). Systemic treatment with an AC1 inhibitor, ST03437 (2.5–10 mg/kg, IP), reduced morphine-induced hyperalgesia in mice. Lumbar intrathecal administration of a viral vector incorporating a short-hairpin RNA targeting *Adcy1* reduced morphine-induced hypersensitivity compared to control mice. In contrast, acute morphine antinociception, along with thermal paw withdrawal latencies, motor performance, exploration in an open field test, and burrowing behaviors were not affected by intrathecal *Adcy1* knockdown. Knockdown of *Adcy1* by intrathecal injection also decreased inflammatory mechanical hyperalgesia and increased burrowing and nesting activity after intraplantar administration of Complete Freund’s Adjuvant (CFA) one-week post-injection.

## Introduction

Of the principal intracellular mechanisms thought to produce tolerance and opioid-induced hyperalgesia in the nervous system, increased adenylyl cyclase (AC) expression and activity is a promising lead candidate (Corder et al., 2013). On a cellular level, upon agonist binding to the mu-opioid receptor (MOR), AC is inhibited and the formation of cyclic adenosine monophosphate (cAMP) is decreased. Paradoxically, prolonged agonist stimulation of the MOR can lead to loss of AC suppression, causing increased intracellular activity of AC, thereby increasing intracellular levels of cAMP (Williams et al., 2001). Enhancement of cAMP levels due to prolonged opioid exposure has long been connected to opioid tolerance and opioid dependence in both *in vitro* (Collier and Francis, 1975; Sharma et al., 1975) and *in vivo* studies, particularly in the spinal cord and dorsal root ganglia (DRG) (Crain and Makman, 1987; Makman et al., 1988).

To date, nine membrane-bound AC isoforms (AC1-9) and one soluble isoform (AC 10) have been confirmed in mammalian nervous systems (Sunahara et al., 1996). AC1 is present in the brain, particularly in the cortex, hippocampus, and cerebellum, and historically has been thought to play a large role in learning and memory (Wu et al., 1995; Defer et al., 2000). AC1 is also present in the spinal cord (Wei et al., 2002) and TrkA positive neurons in the DRG of mice (Haupt et al., 2010). A global loss in AC1 activity results in attenuated nocifensive behaviors after formalin hind paw injection and reduces pCREB activation in the superficial dorsal horn of the spinal cord (Wei et al., 2002).

Although all of the underlying mechanisms behind tolerance and opioid-induced hyperalgesia are not currently known, increased AC expression and activity have been suggested to be one of the major causative agents. To date, it is unknown if selectively inhibiting AC1 activity or reducing AC1 expression after chronic MOR stimulation alters the development of opioid tolerance and opioid-induced hypersensitivity. The purpose of this study was to better understand how the activity of AC1 in the spinal cord and dorsal root ganglia contributes to hypersensitivity seen during morphine tolerance, opioid-induced hypersensitivity, and chronic inflammatory pain. To accomplish this, systemic pharmacological inhibition of AC1 or intrathecal delivery of a short hairpin RNA (shRNA) through a viral vector was used to decrease *Adcy1* expression in mice. Mechanical withdrawal thresholds were measured in morphine tolerant and morphine withdrawn mice. Similarly, several evoked and spontaneous behavioral measures were used to determine if *Adcy1* knockdown would also decrease hypersensitivity or improve mobility in a mouse model of inflammatory pain.

## Materials and methods

### Animals

All experimental procedures involving animals were approved and performed under the University of Minnesota Institutional Animal Care and Use Committee guidelines. Adult male and female C57Bl6 mice were obtained via Charles River (5–6 weeks old, Raleigh, NC) having an average weight of ∼25 g (23–31 g). Mice were housed in a facility ranging from 20 to 26°C on a 14 h light/10 h dark cycle with water and rodent chow (Purina 5015) *ad libitum*. Mice were kept in conventional microisolator cages with no more than five animals per cage. The cages contained irradiated corn cob bedding enriched with aspen and cotton nesting materials. After drug administration and CFA injections, mice were monitored for overall wellbeing and any adverse reactions. Mice were acclimated to individual testing apparatuses before behavioral testing. All experiments were conducted during the 14 h day cycle except for nesting behaviors. Mice were euthanized by isoflurane anesthesia (5%) followed by decapitation at the end of the study. A Table of experimental studies in provided in Table 1.

**TABLE 1:** List of behavioral experiments.

Experiment	Experiment description	Treatment conditions	Behavioral tests administered
1	mRNA of adenylyl cyclases in DRG and spinal cord	Systemic morphine or saline twice daily for 5 days (15 mg/kg, sc)	N/A
2	ST034307 Mechanical and thermal antinociception	Vehicle or ST034307 (2.5–10 mg/kg, ip, 100 µl) 15 min post-morphine	Von frey and thermal paw withdrawal
3	ST034307 Escalating morphine tolerance	Systemic morphine twice daily for 5 days (10 mg/kg on day 1 increasing to 40 mg/kg by day 4, sc, 100µl) + vehicle or ST034307 (2.5–10 mg/kg, ip, 100 µl) 15 min post-morphine, vehicle or ST034307 (2.5–10 mg/kg, ip, 100 µl) on Day 5	Von frey paw withdrawal
4	AC1 knockdown *via* intrathecal delivery: morphine antinociception	AAV9-Adcy1-shRNA or AAV9-Scrambl-shRNA delivered intrathecally, morphine doses 5–20 mg/kg, sc, measurements taken 30 min after morphine injection	Von frey paw withdrawal
5	AC1 knockdown *via* intrathecal delivery: escalating morphine tolerance	AAV9-Adcy1-shRNA or AAV9-Scrambl-shRNA delivered intrathecally, Twice daily escalating injections of morphine (10 mg/kg on day 1 increasing to 40 mg/kg by day 4, sc, 100 µl)	Von frey paw withdrawal
6	AC1 knockdown *via* intrathecal delivery: morphine tolerance	AAV9-Adcy1-shRNA or AAV9-Scrambl-shRNA delivered intrathecally, twice daily injections of morphine for 5 days (15 mg/kg, sc)	Von frey paw withdrawal
7	AC1 knockdown *via* intrathecal delivery: CFA model of inflammatory pain	AAV9-Adcy1-shRNA or AAV9-Scrambl-shRNA delivered intrathecally, CFA delivered intraplantar (one hindpaw)	Before CFA: Rotarod performance test, open field testing, thermal paw withdrawal testing, Von frey paw withdrawal (baseline) after CFA: Von frey paw withdrawal. burrowing testing, nesting

In behavioral studies, 5–10 male and female mice were randomly assigned to either a control or ST034307 treatment group. Viral vector studies consisted of two treatment groups, each containing 10 randomly assigned male and female mice. Use of male and female animals is consistent with the National Institute of Health’s Sex as a Biological Variable policy. For all experiments, male mice were tested before female mice and equipment cleaned in between testing of sexes.

### Drugs and delivery

Morphine (Sigma Chemical, St. Louis, MO, United States) was administered through a 100 µl subcutaneous injection in saline. For morphine tolerance experiments, baseline mechanical paw withdrawal testing was performed before and after administration of 15 mg/kg of morphine for 5 days (Liang et al., 2011). Baseline measurements were measured every morning before morphine injection (Pre) to assess opioid-induced hyperalgesia, and 30 min post injection (Post) to assess tolerance. Escalating morphine tolerance was performed similarly, except increasing doses of morphine, starting at 10 mg/kg and increasing 10 mg/kg/day, were administered over 4 days. Day 5 and day 6 thresholds measured ∼18 and ∼42 h, respectfully, after last morphine injection. In each model, morphine was delivered twice per day (∼0800 and ∼1800 h).

ST034307 (6271, Tocris Bioscience, Minneapolis, MN, United States) was dissolved in 10% β-cyclodextrin with 5% DMSO in saline and administered in a 100 µl intraperitoneal injection. ST034307 or vehicle was administered 15 min after morphine in tolerance experiments.

In separate experiments, Complete Freund’s Adjuvant (CFA, F5881, Sigma Chemical, St. Louis, MO, United States) was administered through an intraplantar injection (20 µl, undiluted) into the left hind paw.

### Mechanical paw withdrawal

Mice were acclimated to the testing environment on at least two separate occasions for 30–60 min before formal testing. The testing environment consisted of a mesh floor, allowing access to animal hind paws, and individual clear acrylic chambers. Mechanical paw withdrawal thresholds, in grams, were determined by electronic von Frey testing equipment (Electric von Frey Anesthesiometer, 2390, Almemo^®^ 2450, IITC Life Science, Woodland Hills, CA, United States). The plantar surface of the hind paws was gently pressed with the probe until a nocifensive response (i.e., paw lifting, jumping, and licking) was elicited. Baseline measurements were collected five times from both the right and left hind paw and averaged, with an interstimulus interval of at least 1 minute.

### Adeno-associated virus-mediated Adcy1 knockdown.

Gene knockdown of Adcy1 using shRNA was achieved using AAV9-GFP-U6-m-Adcy1-shRNA. A scrambled vector, AAV9-GFP-U6-scramble-shRNA, was used in a different group of mice as a control (shAAV-251792 and 7045, titer: 1.4 × 10^13^ GC/mL, in PBS with 5% glycerol, Vector Biolabs, Malvern, PA, United States). Viruses were delivered by direct lumbar puncture (10 uL) in awake mice and behavioral assessments were performed 3–8 weeks post-injection (Fairbanks, 2003; Vulchanova et al., 2010). Intrathecal delivery of AAV9 serotypes in live mice yield high efficacy of transduction efficiency in DRG and lumbar spinal cord while yielding sporadic labeling in the cortex and other peripheral tissues (Schuster et al., 2013).

Four weeks post-virus injections morphine efficacy was determined using an escalating dose-response curve (5–20 mg/kg) waiting 30 min after each injection (Klein et al., 2018). Morphine tolerance or escalating morphine tolerance tests were initiated 5 weeks post-virus injections (see *Drugs and Delivery*)*.* CFA was injected into a cohort of mice 7-weeks post inoculation. CFA treated mice underwent a series of behavioral tests 3–4 weeks after virus injections but before CFA administration (see—*Rotarod Performance Test, Open Field Testing, Thermal Paw Withdrawal Testing*) and after CFA administration (*see – Burrowing Testing, Nesting*).

### Rotarod performance test

Agility assessment was conducted using Rotamex-5 automated rotarod system (0254–2002L, 3 cm rod, Columbus Instruments, Columbus, OH, United States). Mice were placed onto a stationary knurled PCV rod suspended in the air. The initial rotation speed of 4 rpm was gradually increased by 1 rpm in 30-s intervals until animals fell off the rod or reached a speed of 14 rpm (300 s). Two tests were administered per animal and averaged.

### Thermal paw withdrawal test

Latencies to a radiant light beam focused on the plantar surface of each mouse hind paw were obtained using a modified Hargreaves Method (Plantar Test Analgesia Meter, 400, IITC, Woodland Hills, CA, United States) (Hargreaves et al., 1988). The average time required to elicit a nocifensive response of at least three measurements per hind paw were recorded. Mice were acclimated on multiple separate occasions to individual acrylic containers on a shared glass floor heated to 30°C 1 week prior to the start of each experiment.

### Open field testing

The open-field testing arena consisted of a 40 × 40 cm box with a white floor and black walls. Animals were placed in the open-field arena, in a room with controlled adjustable lighting, and baseline activity was recorded for 30 min (Sony Handycam, HDR-CX405, Sony Corp., Tokyo, Japan). The distance traveled (cm), time spent immobile (sec), average velocity (cm/s), and the change in orientation angle (degrees) were computed by using data output from the Ethowatcher computational tool software (Laboratory of Bioengineering of the Institute of Biomedical Engineering and the Laboratory of Comparative Neurophysiology of the Federal University of Santa Catarina, UFSC, available: http://ethowatcher.paginas.ufsc.br/) (Crispim Junior et al., 2012).

### Burrowing testing

Two days after the completion of the mechanical testing (one-week after CFA inoculation), AAV9-*Adcy1* and AAV9-scramble mice were subjected to burrowing testing. Mice were acclimated to empty burrowing tubes for ∼2 h on at least two separate occasions before formal testing. The burrows were made from a 6 cm diameter plastic pipe and 5 cm machine screws were used to elevate the open end by 3 cm (Deacon, 2012). During testing, each mouse was placed in an individual cage with a burrowing tube containing 500 g of pea gravel. The amount of gravel remaining in the tube after 2 h was used to calculate the total percent of gravel displaced from the burrow.

### Nesting

Mice were individually housed in plastic cages containing cob bedding with food and water *ad libitum* overnight. A single 2” Nestlets™ (Ancare Corp., Bellmore, NY, United States) square was weighed and added to each cage. The next morning (∼14 h) untorn pieces of each nesting square were weighed and the resulting nests were photographed and scored on a 5-point scale as described previously (Deacon, 2012). Briefly the scoring system was: 1 = >90% intact, 2 = partially torn, 3 = mostly shredded but no identifiable nest, 4 = >90% torn but flat nest site, 5 = >90% torn with resulting crater nest. Scores with 0.5 units were used for nests with scores in between the aforementioned intervals. Nesting scores were tabulated 3 days after CFA injections.

### Tissue collection and mRNA isolation

Lumbar spinal cord and L3-L6 DRG tissues harvested from animals were flash frozen in liquid nitrogen and stored at -80°C 8 weeks after virus inoculation. Total mRNA was isolated from tissues using Tri Reagent (T9424, Sigma Aldrich, St. Louis, MO, United States) and RNeasy Mini Kit (Qiagen, Germantown, MD, United States) according to the manufacturer’s protocol with 30 min DNase 1 digestion. Complementary DNA synthesis was performed with 50 ng total mRNA using Omniscript RT Kit (Qiagen, Germantown, MD) and random nonamers (Integrated DNA Technologies, Coralville, Iowa) according to the manufacturer’s protocol.

### Quantitative PCR

Quantitative PCR was performed using SYBR Green I dye with a LightCycler 480 machine (Roche, Branchburg, NJ, United States). The cDNA copy number was typically quantified against a ≥5 point, 10-fold serial dilution of a gene-specific cDNA standard (∼5e1 to 5e6 copies/µL) cDNA standards were created using block PCR for one gene (Amplitaq Gold, Applied Biosystems) and purified using Qiaquick (Qiagen). Standards were quantified using a UV-Vis spectrophotometer and DNA copies/µL calculated using the equation: (DNA µg/µl) (µmoles/DNA m. w. µg) (1 mol/1 × 10^6^ µmoles) (6.022 × 10^23^copies/mole). Internal controls included negative RT-PCR samples and comparative expression versus housekeeping genes, 18S and Gapdh. Amplification efficiencies were >1.8 and the targeted ΔCt between two dilutions was around -3.3. Fold expression of each gene of interest was determined by: (mean gene concentration/mean 18s concentration)/(mean gene concentration in saline/mean 18s concentration in saline). See Table 2 for gene-specific primers used.

**TABLE 2:** Gene specific primers used for qRT-PCR. The NCBI gene accession number, resulting base pair length, and both the forward and reverse primers for each gene for qRT-PCR analysis.

Gene	NCBI number	Length	Forward	Reverse
*18s*	NR_003278.3	149bp	5′-CGC​CGC​TAG​AAG​TGA​AAT​TCT​T-3′	5′-CAG​TCG​GCA​TCG​TTT​ATG​GTC-3′
*Adcy1*	NM_009622.1	115bp	5′-TGC​AGA​CAT​CGT​GGG​TTT​CA-3′	5′-ACA​GTG​GTT​TTC​GGC​TA-3′
*Adcy2*	NM_153534.2	140bp	5′-CTA​AAC​CGA​GTG​CTG​CTG​GA-3′	5′-TTG​AAG​TCC​GGA​ATG​GAG​GC-3′
*Adcy3*	NM_138305.3	199bp	5′-TCT​GGG​GTC​CAA​GAA​GAG​AGA-3′	5′-GAC​CCG​GAA​TTT​GGG​ATT​GTC-3′
*Adcy5*	NM_001012765.4	151bp	5′-TGA​TCG​AGG​CCA​TCT​CGT​TG-3′	5′-TGG​TTG​GCC​AGA​GTG​ACA​TC-3′
*Adcy6*	NM_007405.2	162bp	5′-TGC​GGT​GAG​GGA​GAA​TCA​CT-3′	5′-ACA​CCT​GTT​ACC​TCA​CGC​AC-3′
*Adcy8*	NM_001291903.1	191bp	5′-CCG​CAT​CTA​CAT​CCA​TCG​CT-3′	5′-AGT​AGT​AGC​AGT​CCC​CCA​GG-3′
*Prkaca*	NM_008854.5	96bp	5′-TTT​GCC​AAG​CGT​GTG​AAA​GG-3′	5′-AGC​CTT​GTT​GTA​GCC​TTT​GCT-3′
*Prkacb*	NM_011100.4	122bp	5′-TGC​AGC​CCA​GAT​TGT​GCT​AA-3′	5′-ACC​CGA​AAT​CTG​TGA​CCT​GG-3′
*Rapgef3*	NM_001177810.1	145bp	5′-GGA​AGT​GCA​TGA​GCT​GAC​CC-3′	5′-CAC​CTG​GTG​GAT​CCT​GTT​GAA​G-3′
*Rapgef4*	NM_001204165.1	96bp	5′-TCC​AAG​AGC​TGC​CTC​CAT​TG-3′	5′-GAA​TCA​ACG​TCC​CTC​AGA​AT-3′
*Gapdh*	NM_001289726.1	85bp	5′-TGA​CCT​CAA​CTA​CAT​GGT​CTA​CA-3′	5′-CTT​CCC​ATT​CTC​GGC​CTT​G-3′

### AC1 enzyme-linked immunosorbent assay

Quantitative measurement of AC1 in spinal cord tissue was performed using the Mouse Adenylate cyclase type 1 (Adcy1) ELISA Kit (RK08031, ABclonal, Woburn, MA, United States) kit according to manufacturer instructions. Briefly, spinal cords were homogenized in RIPA lysis buffer (100 mM Tris, 150 mM NaCl, 1 mM EGTA, 1 mM EDTA, 1% Triton X-100, 0.5% sodium deoxycholate) with added protease (P8340 and phenylmethylsulfonyl fluoride, Sigma) and phosphatase (P0044 and P5726, Sigma) inhibitors. Spinal cord homogenates were centrifuged and further diluted in Sample Diluent provided by ABclonal. After incubation in Biotin Conjugate Antibody, Strepavidin-HRP, and TMB substrate, each well was read in microplate reader (Biotek Instruments, Santa Clara, CA, United States) at 450 nm with a wavelength correction at 570 nm. Samples were compared against an 8-point standard calibration provided by ABclonal.

### Microscopy

Histological sections were taken from spinal cord, DRG, and sciatic nerves to verify the delivery of the AAV9 virus within the lumbar intrathecal space 8-weeks post inoculation. Verification of virus inoculation was visible by the presence of green fluorescent protein (GFP). Sections (10 uM, Leica CM3050) were mounted onto electrostatically charged slides and images were collected using a Nikon TiS Microscope and associated software.

### C-fiber compound action potentials

Compound action potentials (CAPs) were measured from both left and right desheathed sciatic nerves from AAV9-GFP-U6-m-Adcy1-shRNA and AAV9-GFP-U6-scramble-shRNA8 weeks after intrathecal injection. Sciatic nerves were dissected from the hind limbs of mice and recordings were performed the same day. Each nerve was mounted in a chamber filled with superficial interstitial fluid composed of 107.7 mM NaCl, 3.5 mM KCl, 0.69 mM MgSO_4_, 26.2 mM NaCO_3,_ 1.67 mM NaH_2_PO_4_, 1.5 mM CaCl_2_, 9.64 mM Na^+^ gluconate, 5.5 mM d-glucose, and 7.6 mM sucrose, pH 7.4 (bubbled with 95% O_2_, 5% CO_2_). Electrical stimulation was performed at a frequency of 0.3 Hz with electric pulses of 100-µs duration at 100–10,000 µA delivered by a pulse stimulator (2100, AM Systems, Carlsborg, WA, United States). Evoked CAPs were recorded with electrodes placed ∼5 mm from the stimulating electrodes. Dapsys software was used for data capture and analysis (Brian Turnquist, Bethel University, St. Paul, MN, United States, www.dapsys.net). The lowest stimulus producing a detectable response in the nerve was determined as the threshold stimulus and the peak amplitude determined when the response no longer increased in amplitude. The conduction velocity was calculated by dividing the latency period, the time from stimulus application to neuronal initial response, by the stimulus-to-recording electrode distance. The sciatic nerves were collected in mice previously used in the morphine escalating dose-response curve studies.

### Data analysis

Data were collected by personnel blinded to the animal’s condition and treatment. The appropriate *t*-test, one-way, two-way, or repeated-measures ANOVA followed by either Dunnett’s or Bonferroni’s post hoc analysis was used to determine significance for mechanical thresholds, thermal latencies, gene expression, burrowing, open field testing, rotarod assessments, and CAP recordings. A Mann–Whitney *U* test was used for nesting behaviors. All statistical analyses were carried out using GraphPad Prism version 9 (GraphPad Software, San Diego, CA). Data presented as mean ± SEM unless otherwise indicated with *p* < 0.05 considered statistically significant.

## Results

### Adcy1 mRNA expression is increased in the dorsal root ganglia and spinal cord in mice after chronic administration of morphine

**FIGURE 1:**
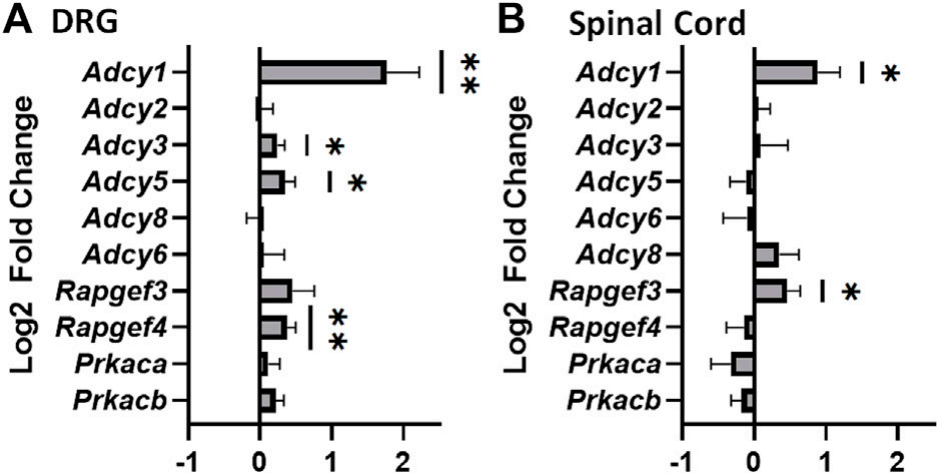
Significant increase in mRNA of adenylyl cyclase 1 in the dorsal root ganglia and spinal cords of mice that have undergone chronic morphine administration. Mice were given systemic morphine or saline twice daily for 5 days (15 mg/kg, sc). **(A)** Dorsal root ganglia (DRG) from morphine treated mice have elevated levels of Adcy1, Adcy3, Adcy5, and Rapgef4 mRNA compared to saline treated mice (one-sample *t*-test, *n* = 11–12/group). **(B)** Spinal cords from morphine treated mice have elevated levels of Adcy1 and Rapgef3 mRNA compared to saline treated mice (one-sample *t*-test, *n* = 14/group). Data are displayed as the Log2 fold change of morphine treated mice over the average of the saline treatment group, ±SEM,*p < 0.05, **p < 0.01.

Chronic agonist exposure of the MOR decreases inhibitory intracellular responses and increases adenylyl cyclase/cyclic-AMP activity (Williams et al., 2013). We attempted to confirm these findings by analyzing the change in mRNA expression of various *Adcy* isoforms in nervous system tissues of morphine tolerant mice. Of the isoforms examined, an increased expression of *Adcy1* is seen in DRG and spinal cord (Figure 1). This suggests AC1 may play a role in morphine tolerance in both the central and peripheral nervous systems. *Adcy3*, *Adcy5*, and *Rapfgef4* (protein: Epac2) mRNA were also elevated in dorsal root ganglia, while *Rapgef3* (protein: Epac1) was also elevated in the spinal cord.

### Systemic ST034307 administration attenuates morphine tolerance and withdrawal

**FIGURE 2:**
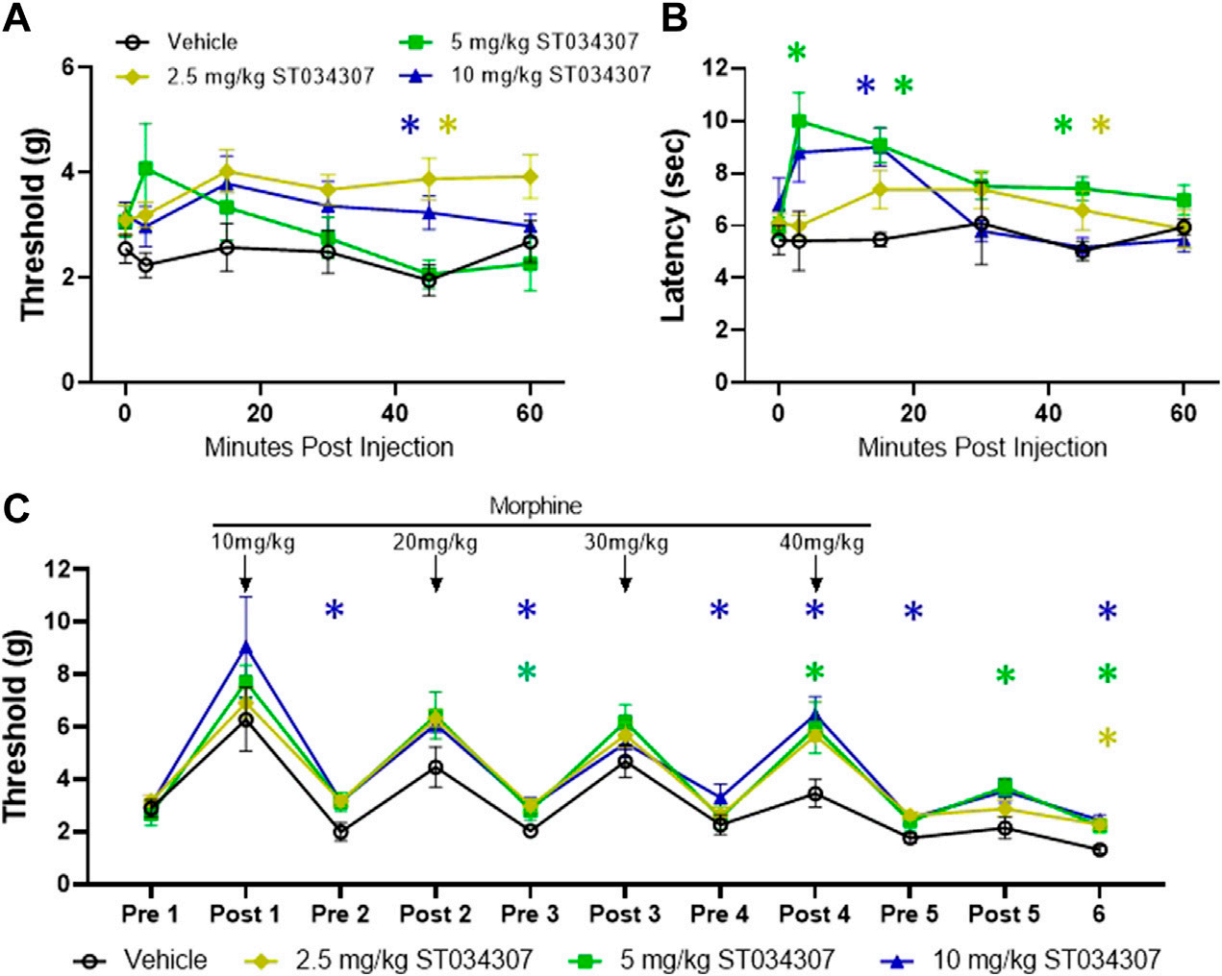
ST034307 produces mechanical and thermal antinociception and attenuates morphine induced hyperalgesia **(A)** Mechanical paw withdrawal thresholds and thermal latencies **(B)** between vehicle and ST034307 treated mice. Significant increases in paw thresholds and latencies were seen between vehicle and ST034307 treated mice (repeated measures ANOVA with Dunnett’s post hoc test vs. vehicle, F (3, 31) = 3.691, *p* = 0.0221 and F (3, 29) = 5.460, *p* = 0.0042, respectively). **(C)** To induce escalating morphine tolerance, mice received twice daily injections of morphine (10 mg/kg on day 1 increasing to 40 mg/kg by day 4, sc, 100 uL) along with an injection of either vehicle or ST034307 (2.5–10 mg/kg, ip, 100 uL) 15 min post-morphine. Baseline measurements were measured every morning before morphine injection (Pre) and 30 min post injection (Post) with day 5 and day 6 thresholds measured ∼18 and ∼42 h, respectfully, after last morphine injection (repeated measures ANOVA with Dunnett’s post hoc test vs. vehicle, F (3, 32) = 8.424, *p* = 0.0003). Asterisk indicates statistical significance at each individual time point (*p* < 0.05). Data presented as mean ± SEM. Data presented as mean ± SEM with an *n* = 5 (vehicle) or 10 (2.5–10 mg/kg ST034307)/group.

To further understand the physiological role of AC1 during tolerance and withdrawal with chronic morphine administration, pharmacological and gene knock-down strategies were implemented with behavioral assays. Previous research demonstrated ST034307 acts as an AC1 inhibitor and as an analgesic in a mouse chronic inflammatory pain model (Brust et al., 2017). In both mechanical and thermal nociceptive tests, the peak threshold and latency measurements increased after intraperitoneal administration of ST034307 (Figures 2A,B). A significant increase in thermal latency was seen between vehicle and ST034307 over time (Figure 2B). The peak antinociceptive action of ST034307 occurred around ∼15 min post-injection, but this effect is still fairly weak in naïve mice.

To determine if ST034307 attenuates morphine tolerance and opioid-induced hypersensitivity *in vivo*, mice were subjected to twice daily morphine injections (10 mg/kg on day one increasing 10 mg/kg each day to a final concentration of 40 mg/kg, sc) in combination with either an injection of vehicle or ST034307 (Figure 2C). Mechanical thresholds were measured before the start of injections and 30 min post morphine injection to measure opioid-induced hypersensitivity and morphine tolerance, respectively. ST034307 increased paw withdrawal thresholds pre-morphine administration, compared to vehicle injected animals, indicating the pharmacological inhibition of AC1 can decrease morphine-induced hyperalgesia. A mild effect on morphine tolerance was also observed as ST034307 increased paw withdrawal thresholds after morphine administration on Day 5 compared to vehicle controls.

### Intrathecal knockdown of Adcy1 attenuates morphine tolerance and opioid-induced hypersensitivity

**FIGURE 3:**
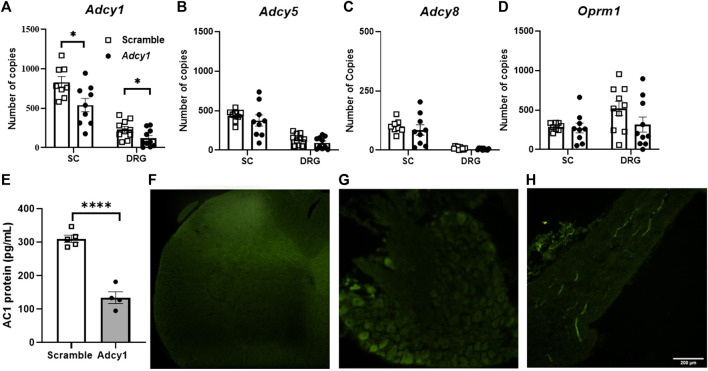
Intrathecal injection of small interfering RNA sequences targeting AC1 reduces Adcy1 mRNA in spinal cord and DRG of mice. **(A)** Significant reduction of Adcy1 mRNA copies present in mice injected with AAV9-ADCY1-shRNA-GFP (Adcy1) vs. AAV9-Scrambl-shRNA-GFP (Scramble; unpaired t-tests, *p < 0.05). No significant changes are seen in Adcy5 **(B)**, Adcy8 **(C)**, or Oprm1 **(D)** mRNA expression. *n* = 8–10/group. **(E)** Protein levels of AC1 collected from spinal cord are lower in AAV9-ADCY1-shRNA-GFP (Adcy1) vs. AAV9-Scrambl-shRNA-GFP injected mice (unpaired *t*-test, *p* < 0.0001), *n* = 4–5/group. Injections were verified by Immunofluorescence analysis in the spinal cord **(F)**, DRG **(G)**, and sciatic nerves **(H)**. Scale bar = 200uM. Data presented as mean ± SEM.

A shRNA targeting *Adcy1* was used to reduce *Adcy1* expression within the peripheral nervous system and spinal cord via intrathecal injection. To ensure the shRNA knockdown strategy of the AAV9-*Adcy1* was successful, spinal cords and DRG were collected for qPCR after the conclusion of behavioral tests. The mRNA copy numbers of *Adcy1* were significantly reduced in AAV9-*Adcy1* mice in both the spinal cord (Figure 3A, unpaired *t*-test, *p* = 0.0201) and DRG (Figure 3A, unpaired *t*-test, *p* = 0.0370) compared to the AAV9-scramble mice. Changes to the expression levels of *Adcy5*, *Adcy8*, and *Oprm1* were also analyzed, but no significant differences were seen for any of these genes in either tissue (Figures 3B–D). Protein levels of AC1 in the spinal cord were also confirmed to be decreased using an ELISA assay (Figure 3E). Florescence microscopy also indicates the injection location was successful in these experiments (Figures 3F–H).

**FIGURE 4:**
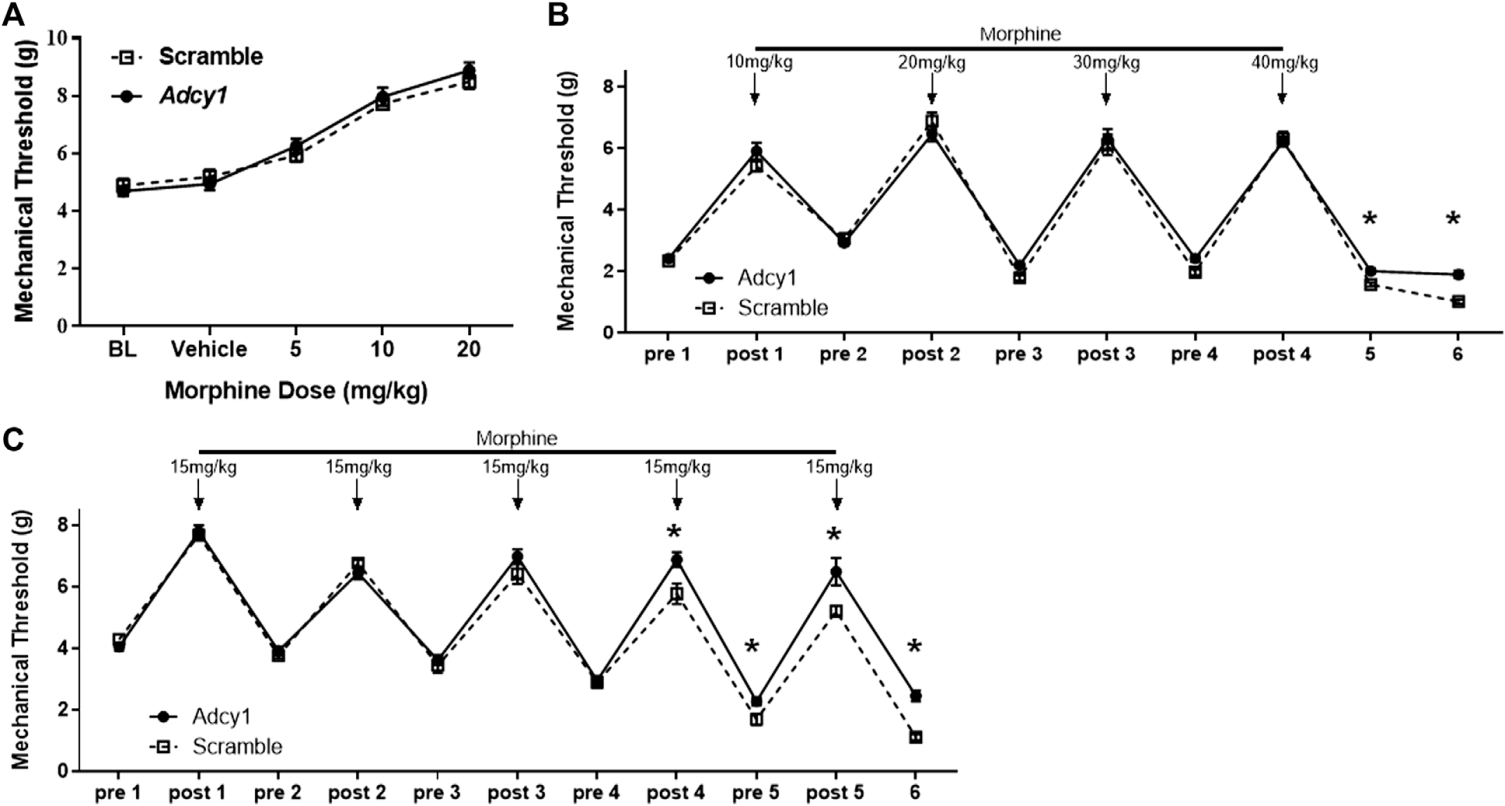
AC1 knockdown *via* intrathecal delivery of AAV9-Adcy1-shRNA partially attenuates morphine induced hyperalgesia in mice. **(A)** Knockdown of Adcy1 mRNA does not impact acute morphine antinociception (5–20 mg/kg, sc, measurements taken 30 min after morphine injection). **(B)** Twice daily escalating injections of morphine (10 mg/kg on day 1 increasing to 40 mg/kg by day 4, sc, 100 µl) induce hyperalgesia that is attenuated in AAV9-Adcy1-shRNA compared to AAV9-Scrambl-shRNA injected mice on Days 5 and 6, ∼18 and ∼42 h after last morphine injection, respectively (repeated measures ANOVA, F (1, 18) = 3.928, *p* = 0.00630). **(C)** Morphine tolerance and hyperalgesia established by twice daily injections of morphine for 5 days (15 mg/kg, sc) are reduced in AAV9-Adcy1-shRNA compared to AAV9-Scrambl-shRNA injected mice (F (1, 18) = 17.61, *p* = 0.0005). Asterisk indicates statistical significance at each individual time point (*p* < 0.05). Data presented as mean ± SEM with an *n* = 10/group.

Since continued agonist stimulation of the MOR increases AC activity, the *Adcy1* knockdown model was hypothesized to show an attenuation of morphine tolerance and opioid-induced hypersensitivity, but not necessarily acute morphine antinociception. An acute morphine dose-response curve indicated AAV9-*Adcy1* and AAV9-scramble mice had similar antinociceptive effects of morphine (Figure 4A). Using an escalating morphine tolerance model, mice were subjected to mechanical testing while given twice daily injections of increasing doses of morphine in saline, starting with 10 mg/kg on day 1 and increasing by 10 mg/kg daily until reaching 40 mg/kg on day 4. AAV9-*Adcy1* mice exhibited significantly higher mechanical thresholds than AAV9-scramble mice pre-morphine administration on days 5 and 6 (Figure 4B). In an alternative model of morphine tolerance, morphine was administered twice daily in saline (15 mg/kg, SQ), and on day 6 mechanical thresholds were taken ∼18 h after the last morphine injection. AAV9-*Adcy1* mice had significantly higher mechanical thresholds compared to AAV9-scramble mice pre-morphine administration on day 5 and day 6, indicating that loss of AC1 reduced morphine-induced hypersensitivity (Figure 4C). Collectively, these data show the knockdown of *Adcy1* not only attenuates the development of morphine tolerance but also the development of opioid-induced hypersensitivity.

### Intrathecal knockdown of Adcy1 improves mechanical hypersensitivity and non-evoked behaviors after complete freund’s adjuvant injection in mice

**FIGURE 5:**
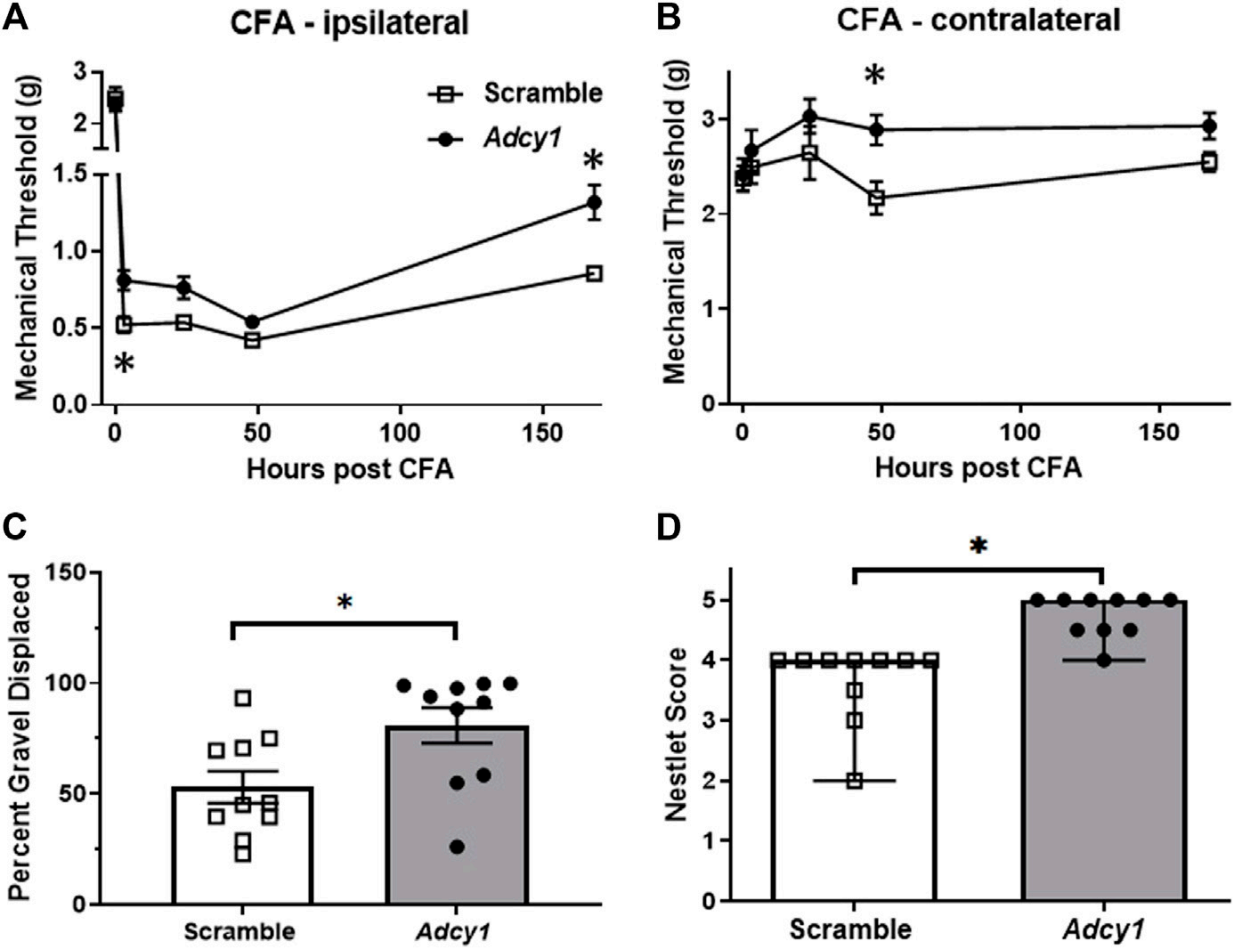
Intrathecal delivery of AAV9-Adcy1-shRNA reduces mechanical hypersensitivity following intraplantar CFA. Mechanical paw withdrawal thresholds were measured before and after a unilateral hind paw injection of 20 µl CFA. **(A)** AAV9-*Adcy1* (●) mice had significantly higher MPW thresholds than AAV9-scramble mice (□) on the injected paw (repeated measures ANOVA with Bonferroni’s *post hoc* test, F (1, 18) = 6.157, *p* = 0.0232), **(B)** and on the CFA uninjected paw (repeated measures ANOVA with Bonferroni’s *post hoc* test, F (1, 18) = 9.148, *p* = 0.0073). **(C)** AAV9-*Adcy1* mice have increased burrowing (unpaired *t*-test, *p* = 0.0188) **(D)** and higher nest building scores compared to control mice (Mann Whitney test, *p* < 0.0001). Asterisk indicates statistical significance at each individual time point (*p* < 0.05), *n* = 10/group. Data in A-C presented as mean ± SEM, data in D presented as median ± 95% confidence interval.

Previous research has demonstrated pharmacological inhibition of AC1 *via* ST034307 could provide analgesia in a mouse model of chronic inflammatory pain (Brust et al., 2017). We performed a similar test to examine CFA analgesic efficacy after *Adcy1-*shRNA treatment 7 weeks after inoculation. AAV9-*Adcy1* mice had significantly higher mechanical thresholds than AAV9-scramble mice on both the CFA injected paw (Figure 5A) and the uninjected hind paw (Figure 5B). This data indicates knockdown of *Adcy1* does provide some analgesic efficacy in the chronic inflammatory pain model one-week post-CFA administration.

A significant increase in gravel displacement was seen between the AAV9-*Adcy1* and control mice (Figure 5C). A significant increase in nesting scores was also seen in AAV9-Adcy1 compared to AAV9-scramble mice (Figure 5D). Altogether, this data indicates the level of ongoing pain or discomfort produced by CFA may be decreased after AC1 knockdown and the loss of AC1 signaling may contribute to greater functional motility during chronic pain.

### Knockdown of Adcy1 does not alter mobility, thermal nociception, or sciatic nerve conduction in mice

**FIGURE 6:**
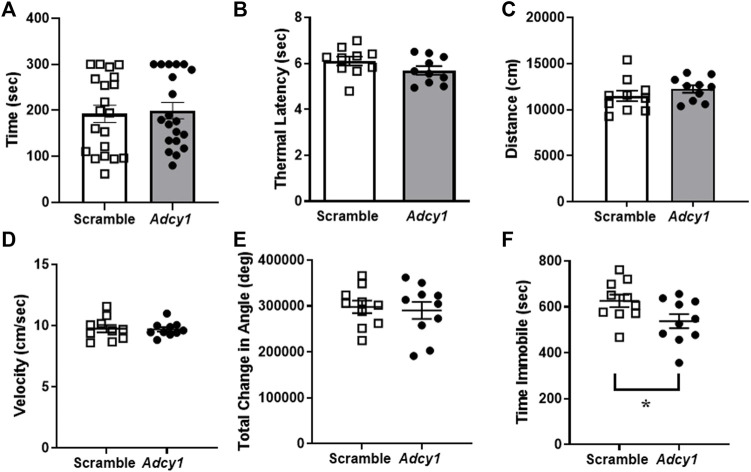
Animal mobility, thermal sensitivity and open field behaviors are not affected in mice with intrathecal knockdown of *Adcy1*. Mice underwent behavioral assessments to gauge mobility and the presence of behavioral deficits after intrathecal injection of AAV9-GFP-U6-m-*Adcy1*-shRNA (●) or AAV9-GFP-U6-scramble-shRNA (□) intrathecal injections. The **(A)** maximum time spent on a rotating rod, **(B)** thermal paw withdrawal latency, open field assessments including the **(C)** distance traveled, **(D)** velocity, and **(E)** total change in angular orientation were not significantly different between treatment groups. **(F)** The time spent immobile in seconds was significantly decreased in AAV9-*Adcy1 compared to* AAV9-scramble mice (unpaired *t*-test, *p* = 0.0433), *n* = 10/group.

**FIGURE 7:**
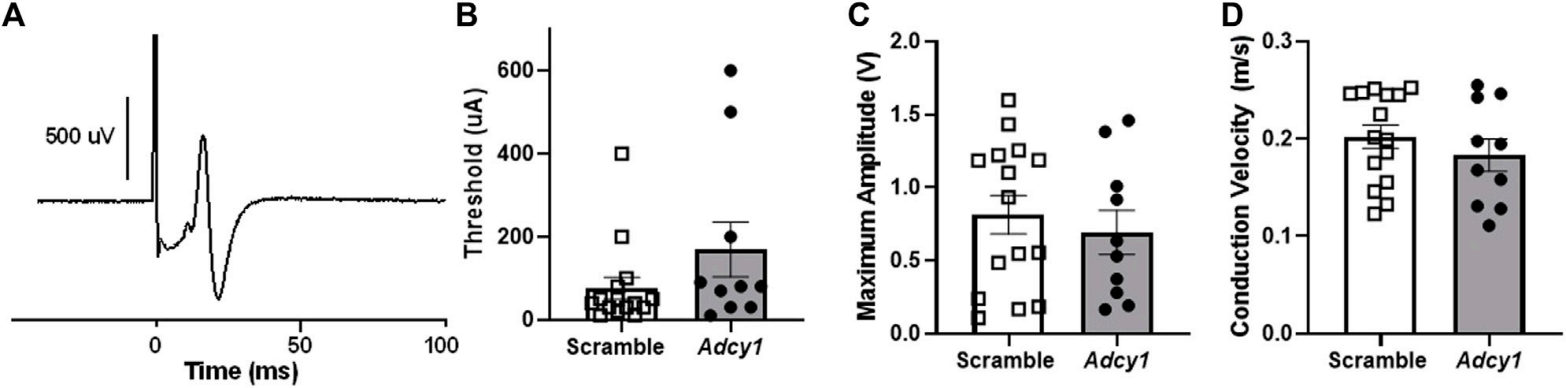
Intrathecal administration of AAV9-GFP-U6-m-*Adcy1*-shRNA (●) or AAV9-GFP-U6-scramble-shRNA (□) does not alter sciatic nerve C-fiber conduction properties. **(A)** Example of a compound action potential (CAP) recording from sciatic nerve of mouse. The **(B)** electrical thresholds, **(C)** maximum CAP amplitude, and **(D)** conduction velocity, were not significantly different between groups. Data presented as mean ± SEM with *n* = 10–20/group.

Mice were subjected to rotarod and thermal paw withdrawal testing 3-weeks post virus injections and open field assessments 4-weeks post virus injections, before CFA administration. For rotarod testing, the total time on rotarod (Figure 6A) were not significantly different between AAV9-scramble and AAV9-*Adcy1* mice. No significant difference was seen between AAV9-scramble and AAV9-*Adcy1* mice during thermal testing (Figure 6B). In open field tests, no significant difference was seen between AAV9-scramble and AAV9-*Adcy1* mice in distance traveled (Figure 6C), velocity (Figure 6D) or change in orientation angle (Figure 6E). However, a small yet significant difference was seen in time spent immobile (Figure 6F; unpaired *t*-test, *p* = 0.0433) indicating AAV9-*Adcy1* mice spent less time stationary compared to AAV9-scramble injected mice. This data indicates the AAV9-*Adcy1* shRNA does not cause any major mobility changes in mice. Lastly, the downregulation of *Adcy1* did not have any impact on thresholds, amplitude, or conduction velocity of C-fiber CAPs (Figures 7A–D).

## Discussion

The present study investigated the role of AC1 with regard to opioid tolerance, opioid-induced hyperalgesia, and inflammatory pain in mouse models. In our study, *Adcy1* was elevated in the DRG and spinal cord of mice after chronic morphine exposure. Our behavioral results indicate pharmaceutical inhibition of AC1 using ST034307 reduced opioid tolerance and attenuated morphine-induced hypersensitivity after increasing opioid administration. Intrathecal knockdown of *Adcy1* using a viral strategy was also effective at reducing morphine-induced hyperalgesia and withdrawal. The loss of *Adcy1* expression increased mechanical paw withdrawal thresholds, and improved burrowing and nesting behaviors after CFA intraplantar injection.

The mechanisms driving chronic pain are thought to be associated with opioid tolerance as both phenomena may arise from similar changes in intracellular signaling pathways in the peripheral and/or central nervous systems (Joseph et al., 2010). The rationale for using both morphine and inflammatory pain models in our study were that 1) Chronic morphine treatment has been previously shown to produce a hypertrophied state of AC1, AC6, and AC8 *in vitro* (Avidor-Reiss et al., 1997) and 2) Several AC isoform-selective pharmacological inhibitors have been developed, particularly for AC1, and appear to attenuate chronic pain in mice (Brust et al., 2017; X.-H. Li et al., 2020; Vadakkan et al., 2006; H. Wang et al., 2011). In chronic pain and opioid-tolerant states, increased AC activity is thought to contribute to enhanced neurotransmission of nociceptive circuits at several levels including the brain (Liauw et al., 2005; Zachariou et al., 2008), spinal cord (Wei et al., 2006), and primary afferents (Yue et al., 2008; Bavencoffe et al., 2016). Opioid tolerance and dependence can be enhanced in several mouse models after CFA treatment, indicating that some synergy is occurring between these two conditions (Liang et al., 2006). In our current and previous studies, systemic administration of ST034307, a small molecule inhibitor of AC1, did not greatly increase mechanical or thermal paw withdrawal thresholds in naïve animals, yet reduced morphine tolerance and hyperalgesia in CFA treated mice. (Brust et al., 2017). This further corroborates the idea that adenylyl cyclase activity in uninjured or morphine-naïve animals may be fairly low.

Hypersensitivity and hyperalgesia seen in chronic pain and drug-induced hypersensitivity most likely occur on multiple levels along sensory transmission pathways, from peripheral afferents, spinal cord synapses, and connectivity across midbrain and cortical cells. To determine if localized downregulation of AC1 in the spinal cord and primary afferent neurons could attenuate opioid tolerance and inflammatory chronic pain, an intrathecal viral knockdown approach was used with shRNA specifically targeted to *Adcy1*. Static dosing of morphine (15 mg/kg, 2x daily, 5 days) and escalating doses of morphine over 4 days (10–40 mg/kg, 2x daily) both resulted in enhanced baseline mechanical sensitivity. After intrathecal administration of AAV9-*Adcy1,* mice had higher mechanical paw withdrawal thresholds before (pre) and after (post) morphine administration compared to control mice. Our data agree with previous studies, as a global loss of either/both AC1 or AC8, appear to have a role in attenuating morphine tolerance and withdrawal (Villacres et al., 1998; Wang and Zhang, 2012). Additionally, AC1 and AC8 knockout mice have increased thermal latencies during the first few days of morphine tolerance testing as well as decreased withdrawal behaviors (Li et al., 2006; Zachariou et al., 2008).

In contrast, baseline mechanical sensitivity and acute morphine antinociception were not changed in AAV9-*Adcy1* mice compared to control mice. This distinction between a lack of antinociception after acute morphine delivery and a significant enhancement of paw withdrawal thresholds after chronic morphine administration is important, indicating adenylyl cyclase hypertrophy occurs after repeated stimulation of the MOR, and not after a single dose of an opioid, which has been a proposed paradigm for many years (Williams et al., 2001). Collectively, this suggests a reduction in the activity or function of AC1 may represent a novel analgesic target in addition to improving opioid withdrawal in patients taking chronic opioids.

AC1 and AC8 have also been linked to the development of both acute and chronic persistent inflammatory pain (Vadakkan et al., 2006; Wei et al., 2006; Griggs et al., 2017; Griggs et al., 2019). Baseline thermal and mechanical sensitivities are not altered in AC1^−/−^ mice, but responses after formalin and CFA are decreased compared to wild-type mice (Wei et al., 2002). In a separate study, the loss of AC1, but not AC8, decreased nocifensive responses to formalin (Vadakkan et al., 2006). Systemic delivery of pharmacological inhibitors of AC1 reduces hypersensitivity in neuropathic and inflammatory pain models in mice (Brust et al., 2017; Wang et al., 2011), consistent with knock-out mouse studies. In a mouse model of inflammatory pain, AAV9-*Adcy1* mice also had higher mechanical paw withdrawal thresholds compared to control mice 3 h and 7 days after CFA injection. The lack of analgesia seen during the initial phases after CFA administration (24–48 h), could be due the deferred hypertrophy of adenylyl cyclases after tissue injury, or the delayed role of adenylyl cyclases in enhanced transcription of pro-inflammatory molecules, taking several days to manifest (Tarnawski et al., 2018). The initial change in mechanical thresholds seen at 3 h post-CFA was surprising, as a previous study failed to demonstrate a pre-emptive effect in a rodent model of neuropathic pain with adenylate cyclase inhibitor administered before injury (Liou et al., 2009). Another previous study using the AC1-specific inhibitor NB001 indicates that inhibition of hyperalgesia after CFA administration was seen 3 days after inoculation (Zhou et al., 2021).

It is possible after tissue damage, stimulation of cAMP and PKA due to adenylyl cyclase activation, promotes hyperalgesia by initially increasing levels of molecules such as PGE_2_ in the early phases of injury (Aley and Levine, 1999). Activation of protein kinase A (PKA) in chronic pain has also been associated with subsequent phosphorylation of a transcription factor, cAMP response element-binding protein (CREB). CREB is responsible for the transcriptional regulation of a large number of proteins and peptides implicated in heightened nervous system activity (e.g. c-Fos, BDNF, tyrosine hydroxylase, etc.) during inflammation and during chronic opioid administration. The impact of AC1 inhibition could be delayed due to the role of CREB or other transcription factors involved in pain chronification that take a much longer time to develop (Tao et al., 2019). Attenuation of mechanical hyperalgesia was also seen on the contralateral (uninjected) hind paws. During chronic pain states, anatomical sites nearby also become sensitized to painful or non-painful stimulation (Chen et al., 2014; Griffioen et al., 2015). The inhibition of AC1 could also help attenuate pain sensitization beyond the primary zone of injury through changes in functional plasticity that occurs in contralateral sensory nerves following the induction of inflammation (Kelly et al., 2007) that may not follow the same time course as the ipsilateral side. AC1 inhibition most likely does not impact the progression of inflammation directly, as other studies have shown that NB001, does not impact CFA-induced knee joint structural destruction nor mono-sodium urate-induced edema of the ankle joint (Tian et al., 2015; Liu et al., 2021). Alternatively, those results may be explained by the smaller overall level of the *Adcy1* knockdown in our studies when compared to global AC1 knockout mice (Wei et al., 2002).

Spontaneous pain and animal wellbeing after initiation of chronic pain are less frequently investigated than evoked measures, so our studies incorporated alternative testing measures including burrowing and nesting behaviors innate to rodents. Burrowing and nesting tests have been used to evaluate spontaneous pain or tonic pain in several models of chronic pain in rodents (Gaskill et al., 2013; Muralidharan et al., 2016). We found small differences between AAV9-*Adcy1* and AAV9-scramble mice in burrowing behaviors after CFA injection. A similar difference was also seen in the nesting scores after CFA induction, as AAV9-*Adcy1* mice demonstrated significantly higher nesting scores than control mice. In previous studies, burrowing behavior is reduced in CFA inflammatory pain models in rats and can be reversed by ibuprofen (Andrews et al., 2012). Nesting behaviors are reportedly attenuated after CFA injection in mice which can be reversed by ketoprofen or low doses of morphine (Negus et al., 2015). Notably, no significant differences were detected between AAV9-*Adcy1* and AAV9-scramble mice in either the rotarod, thermal paw withdrawal latencies, or open field-testing parameters, indicating intrathecal knockdown of AC1 does not affect acute thermal pain thresholds or affect general ambulatory behaviors. Transcriptional knockdown of AC1 in the sciatic nerves of mice was not specifically measured, however, there were no significant changes in C-fiber compound action potential properties. These data indicate behavioral changes seen in the AAV9-*Adcy1* animals may be restricted to the spinal cord and/or DRG, or loss of AC1 function does not impact axonal propagation of C-fiber action potentials.

Opioids are one of the most common analgesics used to alleviate pain in the clinic and function by inhibiting neuronal signal transmission. Individuals with chronic pain, including inflammatory pain, use opioids daily for pain management, causing the development of analgesic tolerance, leading to dosage escalation. Whether the increased opioid requirement is caused by the decreasing analgesic efficacy of the drug (i.e., tolerance), or an increase in spontaneous pain, or lowering the nociceptive threshold, the clinical effect is the same (Hayhurst and Durieux, 2016). Furthermore, if the patient ceases therapy, there is a possibility of withdrawal and hypersensitivity, increasing the likelihood of opioid dependence and abuse situations. New therapies that target the interaction of nociceptive signaling and opioid exposure have not emerged, but would provide an opportunity to not only reduce chronic pain but also potentially ease opioid tolerance and/or dependence. In conclusion, this study suggests AC1 may represent a novel pharmaceutical target for the reduction of chronic pain and the attenuation of opioid-mediated adverse effects. Further research exploring the intracellular targets of AC1 may provide opportunities for new therapeutics in the future.

## Data Availability

The raw data supporting the conclusions of this article will be made available by the authors, without undue reservation.
